# Random forest analysis of midbrain hypometabolism using [^18^F]-FDG PET identifies Parkinson's disease at the subject-level

**DOI:** 10.3389/fncom.2024.1328699

**Published:** 2024-02-07

**Authors:** Marina C. Ruppert-Junck, Gunter Kräling, Andrea Greuel, Marc Tittgemeyer, Lars Timmermann, Alexander Drzezga, Carsten Eggers, David Pedrosa

**Affiliations:** ^1^Department of Neurology, Philipps-University of Marburg, Marburg, Germany; ^2^Clinic for Neurology, University Hospital Gießen and Marburg GmbH, Marburg, Germany; ^3^Center for Mind, Brain and Behavior, Philipps-University of Marburg and Justus-Liebig University Gießen, Marburg, Germany; ^4^Department of Psychiatry, Psychotherapy and Psychosomatics, Vivantes Hospital Neukölln, Berlin, Germany; ^5^Max Planck Institute for Metabolism Research, Cologne, Germany; ^6^Cluster of Excellence in Cellular Stress and Aging Associated Disease (CECAD), Cologne, Germany; ^7^Cognitive Neuroscience, Institute of Neuroscience and Medicine (INM-2), Research Center Jülich, Jülich, Germany; ^8^Multimodal Neuroimaging Group, Department of Nuclear Medicine, Medical Faculty, University Hospital Cologne, Cologne, Germany; ^9^Department of Neurology, Knappschaftskrankenhaus Bottrop, Bottrop, Germany

**Keywords:** Parkinson's disease, imaging biomarker, machine learning, random forest, metabolic imaging

## Abstract

Parkinson's disease (PD) is currently diagnosed largely on the basis of expert judgement with neuroimaging serving only as a supportive tool. In a recent study, we identified a hypometabolic midbrain cluster, which includes parts of the substantia nigra, as the best differentiating metabolic feature for PD-patients based on group comparison of [^18^F]-fluorodeoxyglucose ([^18^F]-FDG) PET scans. Longitudinal analyses confirmed progressive metabolic changes in this region and, an independent study showed great potential of nigral metabolism for diagnostic workup of parkinsonian syndromes. In this study, we applied a machine learning approach to evaluate midbrain metabolism measured by [^18^F]-FDG PET as a diagnostic marker for PD. In total, 51 mid-stage PD-patients and 16 healthy control subjects underwent high-resolution [^18^F]-FDG PET. Normalized tracer update values of the midbrain cluster identified by between-group comparison were extracted voxel-wise from individuals' scans. Extracted uptake values were subjected to a random forest feature classification algorithm. An adapted leave-one-out cross validation approach was applied for testing robustness of the model for differentiating between patients and controls. Performance of the model across all runs was evaluated by calculating sensitivity, specificity and model accuracy for the validation data set and the percentage of correctly categorized subjects for test data sets. The random forest feature classification of voxel-based uptake values from the midbrain cluster identified patients in the validation data set with an average sensitivity of 0.91 (Min: 0.82, Max: 0.94). For all 67 runs, in which each of the individuals was treated once as test data set, the test data set was correctly categorized by our model. The applied feature importance extraction consistently identified a subset of voxels within the midbrain cluster with highest importance across all runs which spatially converged with the left substantia nigra. Our data suggest midbrain metabolism measured by [^18^F]-FDG PET as a promising diagnostic imaging tool for PD. Given its close relationship to PD pathophysiology and very high discriminatory accuracy, this approach could help to objectify PD diagnosis and enable more accurate classification in relation to clinical trials, which could also be applicable to patients with prodromal disease.

## 1 Introduction

Parkinson's disease (PD) is the second most common neurodegenerative disorder (Lau and de Breteler, 2006) and characterized by a spread of α-synuclein containing Lewy bodies and the loss of neuromelanin pigmented neurons in the substantia nigra. The consequential depletion of dopaminergic transmission to lateral nigral projection areas (Kish et al., 1988), and primarily the posterior putamen, results in aberrant striato-thalamo-cortical information processing causing motor symptoms like bradykinesia or rigidity (Albin et al., 1989; DeLong, 1990). Diagnosing the condition can yet be a challenge for physicians, as no reliable biomarker is currently available and only clinical criteria can be used (Postuma et al., 2015). Especially at early stages, when symptoms were present for <5 years, a diagnostic accuracy of only 53% in PD patients has been reported (Adler, 2014). Not only does this limit disease management, but it also underlies the dilemma that neuroprotective therapies are likely to fail if used too late. Therefore, one of the main goals of PD research is to find biomarkers that can be applied easily and early and are as objective as possible (Adler, 2014). Future-oriented concepts claim a biological staging system for PD continuum, whereby degeneration of midbrain dopaminergic neurons represents a crucial, universal feature of the disease.

Currently, there is no causative therapy for PD, but significant efforts have been directed at neuroprotective therapies targeting molecular pathways before disease onset. Nigral neurons are highly energy consuming neural populations relying on effective mitochondria which makes them vulnerable to exhaustion possibly contributing to neurodegeneration (Braak et al., 2006b; Seibyl et al., 2012). When patients experience motor symptoms, typically up to 70% of nigral neurons have already been depleted. Due to lack of applicable α-synuclein tracers, no possibility exists to date for *in vivo* examination of α-synuclein load (Fearnley and Lees, 1991). However, there are indirect measures of nigral dopaminergic cell loss, particularly in the field of molecular imaging. As a surrogate marker for presynaptic dopaminergic activity, semiquantitative analysis of [^123^I]-FP-CIT-SPECT regularly serves as supportive diagnostic tool. To enable diagnosis from a pathophysiological rather than clinical perspective and demonstrate prospects for reducing disease progression through interventions, indicators of biological processes that are immediately applicable and show a strong correlation with established neuropathological markers are urgently needed, especially at early disease stages (Höglinger et al., 2023).

Molecular imaging has been proposed to trace ongoing disease-related processes and subclinical changes. In a recent study applying [^18^F]-fluorodeoxyglucose PET([^18^F]-FDG PET), which uses a labeled glucose analogon, we identified a hypometabolic midbrain cluster as the best differentiating metabolic feature for PD-patients compared to healthy controls (Ruppert et al., 2020). The level of individual hypometabolism was found to match contralateral motor symptoms. Subsequent examinations of a subset of these patients over the course of the disease confirmed progressive metabolic changes in this region which were accompanied by worsened motor symptoms (Steidel et al., 2022). An independent study reported nigral metabolism in PD based on non-high-resolution [^18^F]-FDG PET and demonstrated the great potential of nigral metabolism for differential diagnostics of parkinsonian syndromes (Schröter et al., 2022). Nigral hypometabolism was worse in entities associated with most severe nigrostriatal pathology (Schröter et al., 2022).

Hence, there is a growing body of evidence for the midbrain as an important region to differentiate PD patients from healthy controls based on metabolic group comparisons. Nevertheless, for applications as diagnostic marker, the informative value of the measure for the individual needs to be verified. In this context, machine learning approaches are increasingly applied to evaluate the discriminative accuracy of measures under consideration. Several studies have conducted region-of-interest wise machine learning analysis of [^18^F]-Desmethoxyfallypride PET data extracted from either striatal structures or whole-brain and revealed an accuracy of 59.7% or of about 70% for differentiating between PD patients and atypical parkinsonism (Segovia et al., 2015, 2017a). Studies focusing on [^18^F]-FDG PET as diagnostic marker have rarely been carried out and focused on whole brain scans, or an atlas-based parcellation but did not include the midbrain region despite its crucial role in neuropathology (Wu et al., 2019). In this study, we evaluated midbrain metabolism derived by high-resolution [^18^F]-FDG PET as a diagnostic marker for PD using random forest analysis.

## 2 Materials and methods

### 2.1 Participants

All participants provided informed consent to their data analyses in conformation with the Declaration of Helsinki. The study was confirmed by the local ethics committee (EK12-265) and the Federal Bureau for Radiation Protection. In total, 25 healthy control subjects and 60 patients with clinically established PD were enrolled. Patient recruitment was carried out at the University Hospital of Cologne and affiliated neurology practices, whereas healthy control participants were recruited via advertising. Exclusion criteria were age <40 years, suspected atypical parkinsonian syndromes, advanced parkinsonism, i.e., Hoehn and Yahr stages >3 (Hoehn and Yahr, 1967), dementia, neurological diseases other than PD, and any safety concerns for MRI scanning. In order to exclude patients with dementia, criteria published by the Movement Disorder Society including a neuropsychological test battery and an assessment of the patient's ability to manage daily life (Emre et al., 2007) were applied. The Mini-Mental State Examination (MMSE) was used as cognitive screening tool (Folstein et al., 1975). Clinical examination and functional imaging were conducted at the Max Planck institute for Metabolism Research Cologne and the University Hospital Cologne, Department of Neurology. Patients were examined in the OFF state, defined as a 12-h period without dopaminergic medication (Langston et al., 1992) (72 h in cases of dopamine agonists). Levodopa-equivalent daily dose (LEDD) was calculated for total antiparkinsonian medication based on standard conventions (Tomlinson et al., 2010). Disease severity was quantified by the Unified Parkinson's Disease Rating Scale (UPDRS) part III (Fahn et al., 1987).

Statistical analysis of demographical, clinical and behavioral data was performed in R (R-project for statistical computing, Vienna, Austria). Depending on the assumptions met, parametric or non-parametric tests were performed. Results were considered significant if *p* < 0.05.

### 2.2 [^18^F]-FDG PET acquisition and preprocessing

All PET scans were acquired on an ECAT HRRT-PET-Scanner (CTI) at the Max-Planck-Institute for Metabolism Research in Cologne after overnight fasting and OFF dopaminergic medication. Under standardized conditions (dimmed light, closed eyes, quiet room) subjects were positioned along the kantho-meatal line. Following a transmission scan, 185 MBq of the radioligand was injected intravenously and tomographic images were acquired in dynamic PET scans (60 min). Using camera-specific filters, PET data were corrected for attenuation and scattered radiation, and reconstructed to 207 slices with a 256 × 256 matrix and 1.22 mm voxel size, creating one frame per 10 min. Frames were realigned for motion correction by rigid-body transformation and frames numbered three to six were averaged into one static image for further analysis. The data set used in the presented analysis has been analyzed in previous publications from different research perspectives (Greuel et al., 2020; Ruppert et al., 2020, 2021; Steidel et al., 2022) and once in context of machine learning but with a whole brain approach and in specific combination with metabolomic data (Glaab et al., 2019).

**Figure 1:**
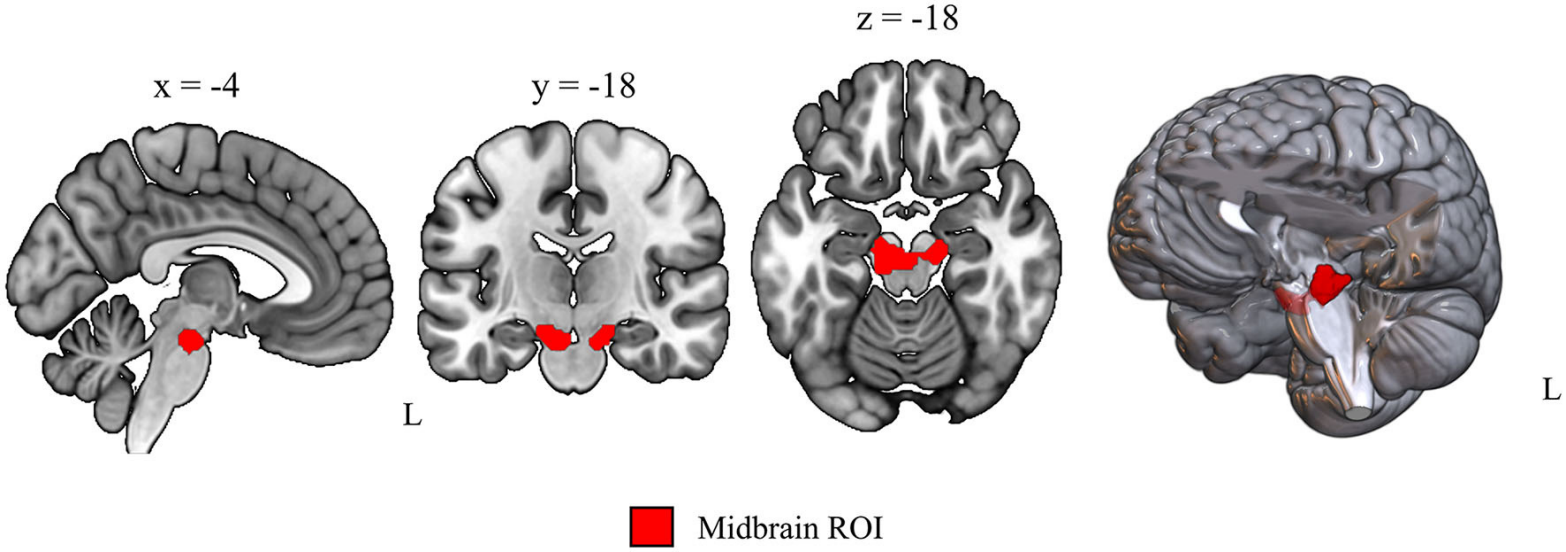
Data-driven region of interest used for voxel-wise uptake extraction. Sagittal, coronal and axial view of the midbrain region of interest obtained by voxel-wise group comparison of [^18^F]-FDG PET scans from 16 healthy controls and 51 PD patients (*p* < 0.01 after FWE cluster level correction, *t* = 6.46, cluster size = 376 voxels).

Static PET scans were spatially normalized into Montreal Neurological Institute (MNI) space in SPM12 (www.fil.ion.ucl.ac.uk/spm/software/spm12, Wellcome Trust Center for Human Imaging, London) using an [^18^F]-FDG PET template for elderly subjects (Della Rosa et al., 2014) and smoothed with a Gaussian kernel of 6 mm full-width at half-maximum (FWHM). The midbrain cluster, reflecting hypometabolic regions in our PD cohort and defining our regions of interest in the current analysis, was derived by a voxel-wise group comparison as specified in our previous work (Ruppert et al., 2020), with the number of included subjects referring to all subjects with [^18^F]-FDG-PET scans here (PD = 51, healthy controls = 16). PET data were proportionally scaled with reference to the global mean as implemented in SPM. Group comparisons were carried out via a general linear model in SPM12. Results were considered significant when *p* < 0.05 after family-wise error (FWE) rate correction at cluster level (Figure 1).

Voxel-wise normalized (proportional scaling) uptake values were extracted from the obtained midbrain cluster (Figure 1) for all subjects with the region of interest toolbox Marsbar (Brett et al., 2002). A class label column was added for supervised machine learning with 0 for healthy control and 1 for PD class. To check whether the approach also performs with a not data-driven region, which would enable easier transferability to independent data sets, we repeated the machine learning analysis with uptake measures from an atlas-based midbrain region (Talairach-Daemon atlas, WFU PickAtlas, RRID:SCR_007378) and with a whole brain gray matter mask [ICBM 2009c non-linear symmetric, FSL (Collins et al., 1999)].

### 2.3 Machine learning analysis

**Figure 2:**
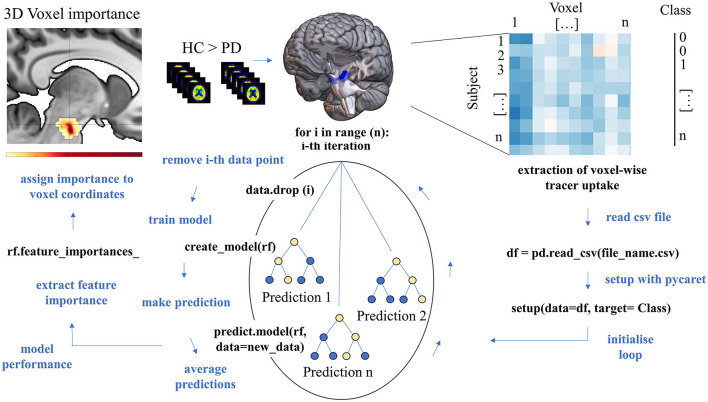
Schematic representation of the data extraction process and random forest analysis. The subcortical region of interest was defined by a between-group comparison of [18F]-FDG PET scans **(middle top)**. Voxel-wise uptake values were extracted from that region for every subject from normalized [18F]-FDG PET scans **(top right)** and subjected to random forest analysis using PyCaret. After importing the data, random forest model was conducted on the data from all but one subject (each subject was left out once) with 10-fold cross-validation. The data set was splitted into a training and validation data set (70:30) to derivate accuracy measures and the class of the removed subject was predicted based on that model. The model's accuracy measures, and each voxel's feature importance were averaged across all runs and reassigned to voxel coordinates to enable 3D representation of voxels with highest importance for class decision.

Extracted uptake values were subjected to machine learning analyses, applying different feature classification algorithms. An adapted leave-one-out cross validation approach with reassignment of the training and validation test set (70:30) at every step was applied for testing robustness of the model for differentiating between patients and controls. First, we compared performance of the most commonly applied machine learning classification algorithms in our data set using PyCaret tool (https://pycaret.org/) in python. Specifically, the following algorithms were tested: Extra Tree Classifier, Naive Bayes, K Neighbors Classifier, Random Forest Classifier, Logistic Regression, Ada Boost Classifier, Light Gradient Boosting Machine, Dummy Classifier, Decision Tree Classifier, Ridge Classifier, Linear Discriminant Analysis, Gradient Boosting Classifier, Support-Vector-Machine—Linear Kernel, and Quadratic Discriminant Analysis. The random forest ensemble algorithm is one of the most widely applied machine learning techniques for classification problems. It is an ensemble learning method, which used a combination of decision trees to make predictions. Each decision tree is trained based on a subset of the data generated by Bootstrap-sampling. A prediction is offered by every decision tree and the final prediction of the model is driven by the majority of votes on the predictions (cf. Figure 2). For hyperparameter tuning, the default option in PyCaret was used which applies a random grid search. Robustness and discriminatory performance of the model across all runs was evaluated in four ways: (1) for model evaluation we averaged performance measures across 67 runs with one of the 67 subjects left out and dividing the remaining 66 in respective training and validation data sets (70:30), (2) in each of these runs a 10-fold nested cross-validation was performed on the training data set with 1-fold serving as validation and 9-fold serving as training data set per one of the 10 validations, (3) in each of the 67 runs, an independent validation data set was used to evaluate model performance by calculating sensitivity and specificity for the resulting confusion matrix, and (4) the percentage of correctly categorized subjects for test data sets (the one not considered in training and validation per run) with reference to movement disorder expert opinion. Included subjects per training and validation data set can be found on our GitHub repository (https://github.com/ruppertm/Midbrain-FDG-PD.git). An evaluation of potential between-group differences in clinical and demographic variables is reported in the Supplementary material.

Feature importance reflects the relevance an individual feature has for correct classification. Feature importance for individual voxels was calculated according to the default settings implemented in PyCaret which refers to the method in the scikit-learn library (mean decrease impurity). Each voxel's coordinates (in all three axes) derived by Marsbar were transformed into MNI-space coordinates using the provided transformation matrix. 3D displays were created in MRIcroGL using the Marsbar coordinates and feature importance values derived via the feature importance analysis above. Spatial colocalization with dopaminergic midbrain nuclei was verified using the Automated Anatomical Labeling version 3 (AALv3) atlas. The code generated to analyze all data is freely available on GitHub (https://github.com/ruppertm/Midbrain-FDG-PD.git).

## 3 Results

### 3.1 Cohort characteristics

[^18^F]-FDG PET scans were available for 51 patients with MRI (66.45 ± 8.53 years, 18 female) and 16 control subjects (64.63 ± 8.33, 9 female) with no significant differences in terms of age, sex and general cognitive performance (cf. Table 1). The included patients were moderately affected with an average UPDRS-III of 25.10 ± 9.54 points and 453.88 ± 244.72 mg LEDD. Detailed information on included participants (mean ± standard deviation) can be found in Table 1. Across all runs, there were no between-group differences in terms of age or motor severity between training and validation data set (Supplementary material).

**Table 1:** Demographic, clinical and behavioral characteristics of the [18F]-FDG PET cohort including all PD patients and healthy controls.

**Groups**	**HC (*n =* 16)**	**PD (*n =* 51)**	**Statistics**	***p*-value**
Age (in years)	64.63 ± 8.33	66.45 ± 8.53	*t* = 0.75	0.455
Female (%)	9 (56.25%)	18 (35.29%)	*X*^2^ = 1.44	0.231
DD (in years)	-	4.56 ± 3.29	-	-
UPDRS III total	-	25.10 ± 9.54	-	-
LEDD (in mg)	-	453.88 ± 244.72	-	-
MMSE	28.94 ± 1.00	28.37 ± 1.82	*W* = 351.5	0.392

Between-group comparison of numeric variables was performed via *t*-tests or Mann-Whitney U tests. Dichotomous variables were compared via chi-square test. DD, disease duration; HC, healthy control subjects; Levodopa equivalent daily dose; PD, Parkinson's disease; MMSE, Mini-Mental Status Examination.

### 3.2 Random forest analysis

#### 3.2.1 Classification based on midbrain [^18^F]-FDG uptake

Across all runs, the random forest algorithm performed best in most cases. Therefore, random forest classifier analysis was applied to evaluate the diagnostic potential of midbrain metabolism in our study. The random forest feature classification of voxel-based uptake values from the 376 voxels spanning midbrain cluster distinguished between the groups with an average sensitivity of 0.91 (Min: 0.82, Max: 0.94) in the validation data set (Table 2). For all 67 runs, in which each of the individuals was treated once as test data set, the test data set was correctly categorized by our model. The separately performed analyses with uptake values from the midbrain atlas region showed slightly lower sensitivity measures, and lower specificity and accuracy (Table 2). Whole-brain analysis revealed a slightly better sensitivity, but worse specificity and accuracy (Table 2).

**Table 2:** Accuracy measures of the random forest classifier model based on [18F]-FDG PET uptake for the data-driven region of interest, midbrain atlas region, and whole brain gray matter.

**Model performance (mean ±SD)**	**Accuracy**	**Sensitivity**	**Specificity**
Midbrain (data-driven)	0.83 ± 0.06	0.91 ± 0.03	0.67 ± 0.14
Midbrain (atlas)	0.82 ± 0.04	0.88 ± 0.05	0.63 ± 0.15
Whole brain gray matter mask	0.76 ± 0.04	0.98 ± 0.03	0.10 ± 0.11

SD, standard deviation.

#### 3.2.2 Feature importance

**Figure 3:**
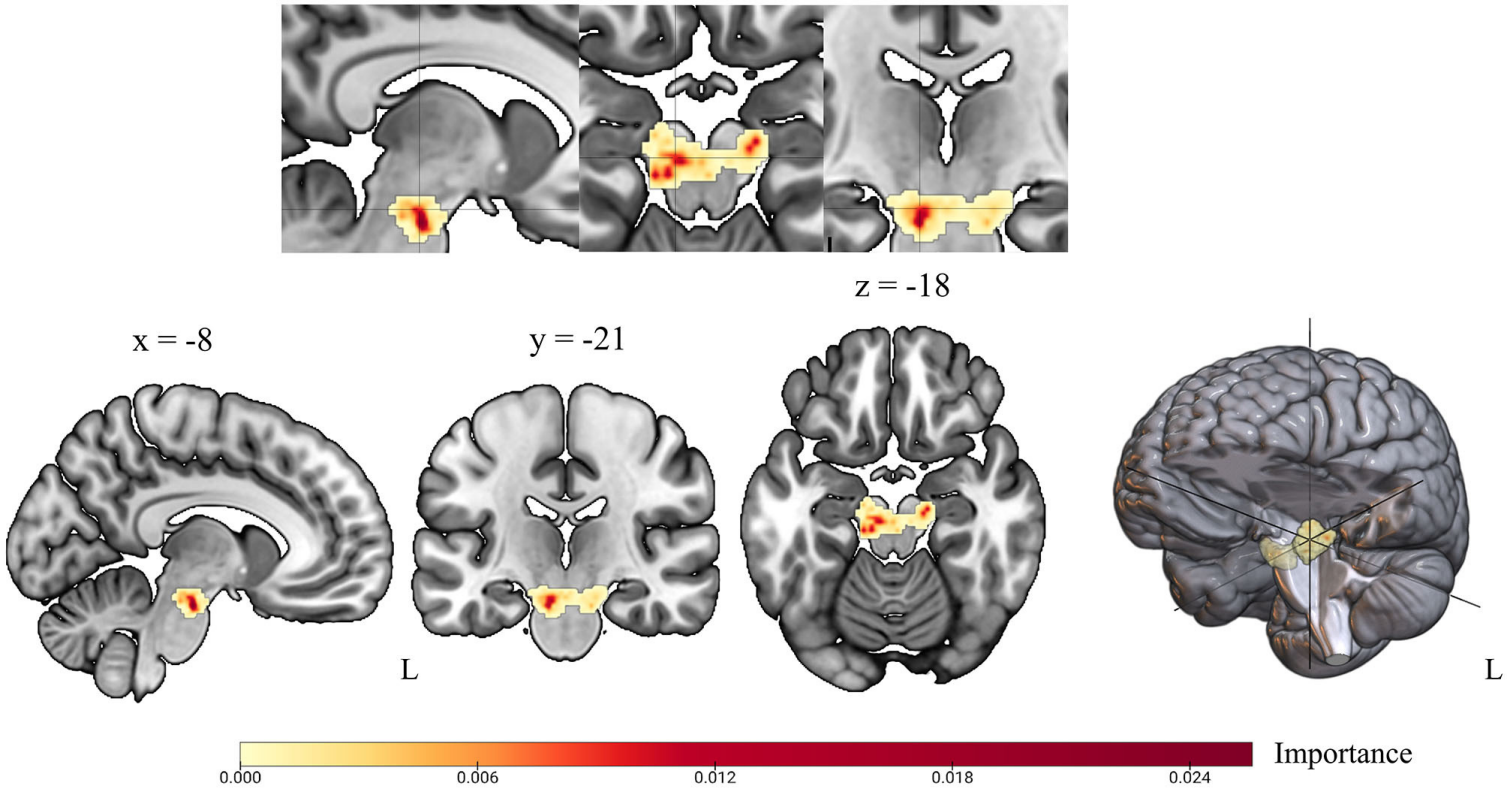
Voxel-wise feature importance for the midbrain region of interest. Feature importance revealed by random forest classifier is shown in color scaled 3D representation for every voxel in the midbrain region of interest. Yellow color indicates minor feature importance; red color indicates high voxel importance for class decision based on random forest classifier.

Since our region of interest is closely related to PD pathophysiology and we included individual voxels as features in our model, the spatial location of features with greatest importance for the class decision was of great interest. The applied feature importance extraction consistently identified a subset of voxels within the midbrain cluster with highest importance across all runs. Among the top voxels with highest importance across all runs were V70 (0.029, MNI: x = −8, y = −20, z = −22) and V148 (0.021, MNI: x = 14, y = −18, z = −20, see Supplementary Table 1 for all values). The two voxels with highest importance were localized in the left ventrolateral tier of the midbrain cluster and next to the atlas region substantia nigra pars compacts (SNpc) from AALv3 atlas (cf. Figure 3 top, Supplementary Figure S1). As indicated by overlay plots in Supplementary Figure S1, there is a spatial overlap between midbrain voxels with high importance and dopaminergic midbrain nuclei. Among the nuclei with a spatial convergence were: left SNpc, left substantia nigra pars reticulata (SNpr), left ventral tegmental areas (VTA), right SNpc, right SNpr, and right VTA. The left SNpc was the atlas region that had the greatest spatial overlap with left-sided voxels of highest importance (dark orange-to-red color) (cf. Figure 3 top, Supplementary Figure S1). Voxels with a feature importance above 0.008 overlapped exclusively with the left SNpc. Right-sided voxels with highest importance were localized more laterally. The separate analysis performed with an atlas-based midbrain region revealed nearly identical coordinates for voxels with highest feature importance (V332 MNI: x = −6, y = −22, z = −20, see Supplementary Figure S2). Including whole brain gray matter [^18^F]-FDG uptake per voxel in a separate analysis, also indicated that our defined region is the most important region for classification (cf. Supplementary Figure S3).

## 4 Discussion

In this study, we demonstrate the diagnostic potential of midbrain [^18^F]-FDG uptake for PD. In a cohort of well-characterized mild-to-moderately affected patients, we showed that it may differentiate between patients and controls with high precision. The presented analyses were motivated by the previous description of the cohort, highlighting the hypometabolic midbrain cluster as the region that exhibited the highest deficit in PD that correlated with contralateral clinical severity (Ruppert et al., 2020) and showed disease-related decline over time (Steidel et al., 2022). In order to evaluate the informative value of [^18^F]-FDG uptake within that region for the individual's classification, a random forest feature classification algorithm was applied with an adapted leave-one-out cross validation approach. Across all runs, the individual test data set was correctly categorized by our model. The applied feature importance extraction consistently identified a subset of voxels within the midbrain cluster with highest importance for class decision across all runs, which spatially overlapped with the left substantia nigra pars compacta. Our results confirm that [^18^F]-FDG uptake in the midbrain is a promising neuroimaging feature with spatial convergence to known pathophysiology that is feasible in the individual patient and can be similarly applied to independent cohorts using midbrain atlas regions.

The loss of dopaminergic cells in the midbrain is a histopathological hallmark of PD and serves as neurobiological correlate of its progression (Damier et al., 1999). However, the significant denervation in the lateral substantia nigra prior to the onset of symptoms in those affected has not been clinically utilized due to a lack of suitable *in-vivo* examination techniques. Notably, particular voxels within our region of interest hold significant importance from a neurobiological viewpoint. There is a spatial overlap of voxels with a feature importance above 0.008 located in the left SNpc, substantiating the hypothesis that the observed hypometabolism might indicate a relationship to degenerating nigral cells or lowered metabolic activity in these naturally energy-demanding cells (Braak et al., 2006a; Seibyl et al., 2012). Corresponding to our earlier analyses, a higher count of voxels with increasing significance for class decision were located in the left midbrain. Our results complement the previous studies in the sense that exactly this region is suitable for the classification of an individual with high precision.

Machine learning techniques are used to identify elusive patterns that are difficult to detect using conventional statistical methods and to test their predictive power at individual level (Peng et al., 2020). Notably, despite certain efforts to apply machine learning to [^18^F]-FDG-uptake for identifying-PD patients (Shen et al., 2019; Wu et al., 2019), none have targeted the midbrain region specifically. Another study has reported the identification of critical diagnostic features in the midbrain based on deep-learning, and claimed that this region, despite its crucial involvement in PD pathophysiology, has not been considered in conventional [^18^F]-FDG PET studies (Zhao et al., 2019). Yet, several parallels might be drawn to previous attempts of applying machine learning to PET data of PD cohorts. Wu et al. (2019) extracted radiomic features from PET images using atlas regions excluding the midbrain (Wu et al., 2019). Shen et al. (2019) followed an approach with Group Lasso Sparse Deep Belief Network (GLS-DBN) for identifying PD based on [^18^F]-FDG PET scans. Both studies report a diagnostic accuracy comparable to our results (Shen et al., 2019; Wu et al., 2019), but do not elaborate the importance of a specific subcortical region that has a close association with the known pathology as our results do. Another study has conducted a machine learning analysis with the here presented [^18^F]-FDG PET data set but focused on whole brain uptake for PD diagnosis. Our region of interest-based approach revealed higher accuracy for the PET modality (Glaab et al., 2019). In line with our findings, Segovia and colleagues also reported a higher diagnostic accuracy with a focus on specific disease-related regions of interest rather than whole brain analysis in a dopaminergic PET study (Segovia et al., 2015, 2017b). A combination of multiple imaging modalities, supported by a specific focus on disease-related regions as in the presented approach, could increase model performance and could be crucial for tracking disease progression. Particularly, our results may be of relevance for efforts of establishing objective markers for a purely biological-based staging system for the disease spectrum, as recently proposed and already established for other neurodegenerative disorders (Chahine et al., 2023; Höglinger et al., 2023). In the latter conceptual framework, degeneration of dopaminergic neurons in the midbrain is a crucial feature evident universally in PD syndromes (Chahine et al., 2023) and present in both presumed retro- and anterograde spreading subtypes. This fact and the recognized significance of FDG-PET patterns in PD (Höglinger et al., 2023) lends our target an important status with potential applicability within the framework.

Based on Schröter et al. (2022)'s findings, our approach may additionally serve to distinguish between atypical Parkinson's syndromes and PD. The fact that the latter study reported similar evidence for midbrain hypometabolism based on not high-resolution PET data suggests that the presented approach is likely to be replicated with standard clinical PET data and therefore easily integrable into clinical practice.

### 4.1 Limitations

One limitation of this study is the small sample size, especially in the healthy control group, which especially contributes to very unbalanced validation data sets. The limited number of controls was a deliberate decision in line with the specifications of the Federal Office for Radiation Protection to include as few healthy subjects as possible. The presence of unbalanced data and a rather small sample size warrants some caution on generalizable conclusions. In particular, unbalanced data may aid in more precise identification of PD patients compared to healthy control subjects' categorization since the individual model was likely trained on a higher number of patients compared to controls. Subsequent studies should therefore include larger sample sizes and equally sized groups. However, the present study reveals initial implications for the approach by applying an appropriate model for unbalanced data. In addition, appropriate techniques like conducting an ensemble of random forest analyses and evaluating the variability of model performance across runs, internal 10-fold cross validation and model evaluation based on respective validation data sets and prediction for one independent subject were taken into account. As a logical consequence of the preliminary work, however, the study provides initial indications, and an interesting proximity to neuropathology with accessibility on subject level, which could also be applicable to patients with prodromal disease in future studies.

A major limitation of the present study is the absence of testing the model on an external cohort, which would supplement the generalizability of the results. We conducted the analysis with high-resolution HRRT PET data to enable tracing back effects on smallest midbrain structures in terms of pathophysiological relevance. We have not tested our approach in an independent sample, as there is no large public dataset of high-resolution PET data. However, a more widespread availability of higher resolution scanners in the future and a multicenter initiative for collecting data may foster possibilities for an independent data set. In addition, future projects could focus on the comparability with lower resolution data as recent studies suggest that our approach might be feasible in non-high-resolution data that are more widely available. Furthermore, our implementation of supervised learning relied on the subjective evaluations of two independent clinical experts in movement disorders, which may not always reflect the ground truth, and should be supported by more objective diagnostic criteria as proposed by biological PD models, including molecular CSF markers, evidence of rapid eye movement sleep behavior disorder (RBD) and dopaminergic imaging, especially in prodromal stages.

### 4.2 Future perspectives

Similar to other studies using machine learning techniques, there is a question about scalability or applicability of this relatively simple measures in independent cohorts. Future studies could validate the approach presented here in early or prodromal stages of the disease, such as patients with RBD, as differences could be expected according to the longitudinally observed midbrain hypometabolism (Steidel et al., 2022). As recent studies highlight a pivotal role for evidence of nigrostriatal degeneration also in the pre-motor phase of the disease, our results may have direct implications for the emerging field of early diagnostics and identifying at-risk persons. The application of such kind of *in-vivo* accessible, objective biomarkers is of greatest interest in context of new therapeutic treatment strategies and paralleled by the development of disease-modifying agents. As longitudinal midbrain changes were demonstrated in mid-stage patients, future studies should verify if midbrain hypometabolism can be identified in prodromal stages like RBD-patients with high-resolution PET. Identifying prodromal biomarkers may be helpful for identifying early disease stages, a crucial element for clinical trials of potential neuroprotective drugs, antibody studies or cell-based therapies.

## 5 Conclusion

Midbrain metabolism measured by [^18^F]-FDG PET is a promising imaging tool for detecting PD-related midbrain degeneration on subject-level. Given its close relationship to PD pathophysiology and very high sensitivity, this approach can index midbrain degeneration and help to establish neurobiological staging systems, addressing the nigrostriatal system.

## Data availability statement

The raw data supporting the conclusions of this article will be made available by the authors, without undue reservation.

## Ethics statement

The studies involving humans were approved by local Ethics Committee University of Cologne, Faculty of Medicine. The studies were conducted in accordance with the local legislation and institutional requirements. The participants provided their written informed consent to participate in this study.

## Author contributions

MR-J: Conceptualization, Data curation, Formal analysis, Investigation, Methodology, Validation, Visualization, Writing – original draft, Writing – review & editing. GK: Conceptualization, Formal analysis, Investigation, Methodology, Software, Validation, Writing – review & editing. AG: Data curation, Investigation, Writing – review & editing. MT: Writing – review & editing. LT: Resources, Supervision, Writing – review & editing. AD: Resources, Writing – review & editing, Funding acquisition. CE: Conceptualization, Project administration, Resources, Validation, Writing – review & editing, Funding acquisition. DP: Resources, Writing – review & editing, Conceptualization, Project administration, Validation.
