# Supplementary material for: Random forest analysis of midbrain hypometabolism using [18F]-FDG PET identifies Parkinson's disease at the subject-level
Source: Front Comput Neurosci. 2024 Feb 7;18:1328699. doi: 10.3389/fncom.2024.1328699 (PMC10879348; doi:10.3389/fncom.2024.1328699)
Supplement: Supplementary file 1 [file Data_Sheet_1.pdf]

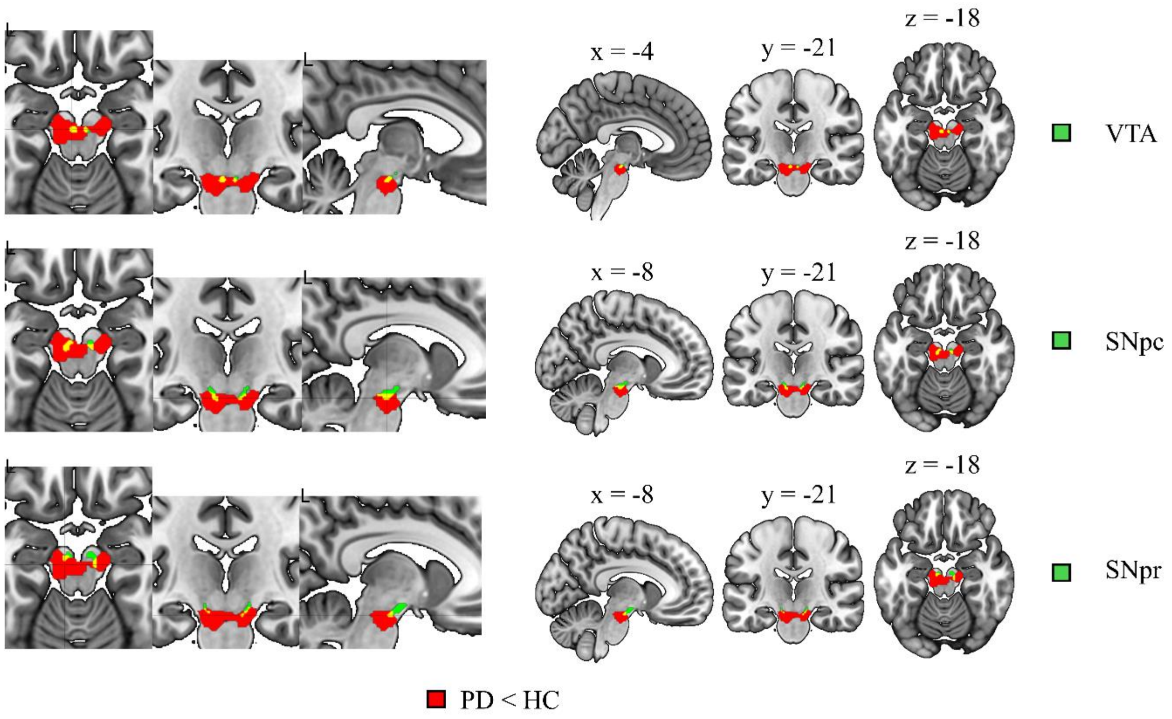

Figure S1: Spatial overlap between the region of interest defined by group comparison of [18F] FDG-PET scans and AALv3 dopaminergic nuclei masks.

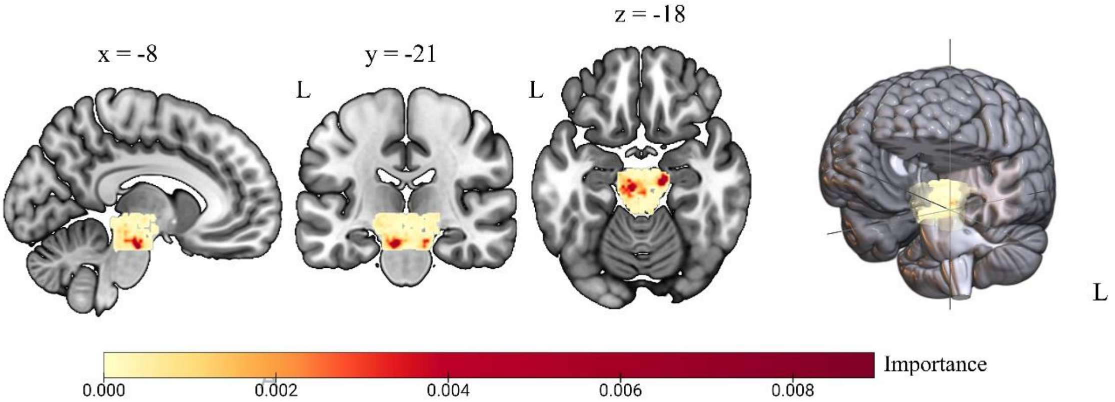

Figure S2: Voxel-wise feature importance for the atlas-based midbrain region of interest (Talairach-Daemon atlas). Feature importance revealed by random forest classifier is shown in color scaled 3D representation of for every voxel in the midbrain region of interest. Yellow color indicates minor feature importance; red color indicates high voxel importance for class decision based on random forest classifier.

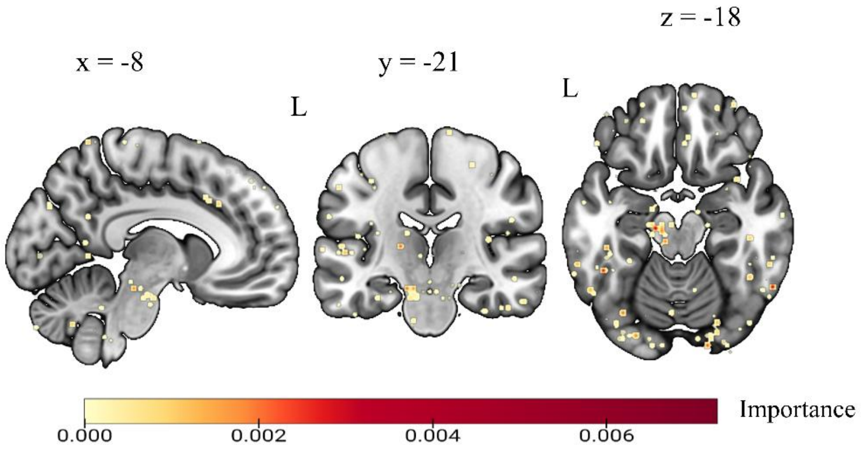

Figure S3: Voxel-wise feature importance for the whole brain grey matter region of interest (Talairach-Daemon atlas). Feature importance revealed by random forest classifier is shown in color scaled 3D representation of for every voxel in the midbrain region of interest. Yellow color indicates minor feature importance; red color indicates high voxel importance for class decision based on random forest classifier.

Table 1: Average feature importance per voxel of the data-driven midbrain region across runs

Feature	Importance
V70	0,029335821
V148	0,020949731
V83	0,020790537
V160	0,019323687
V143	0,017987522
V126	0,016138848
V244	0,014606627
V185	0,013915925
V183	0,013556299
V276	0,013177239
V208	0,01304894
V162	0,012651806
V232	0,012228746
V195	0,01117794
V355	0,011105746
V209	0,010992433
V28	0,010669358
V76	0,009576776
V226	0,009275612
V365	0,008513597
V35	0,008491104
V227	0,008465687
V142	0,008282554
V273	0,008153313
V207	0,008050746
V225	0,007974134
V71	0,007858582
V77	0,007587269
V330	0,007279418
V86	0,006875194
V197	0,006859836
V324	0,00682909
V100	0,00680494
V85	0,00663297
V84	0,006555209
V64	0,00650997
V228	0,006427642
V169	0,00638834
V310	0,006337119
V61	0,006324448
V184	0,006266283
V150	0,006134818
V215	0,006053448
V144	0,005790866
V205	0,005724866
V82	0,005714742
V127	0,005679149
V286	0,00560797
V91	0,005570075
V309	0,005436224

V267	0,005333373
V262	0,005301328
V177	0,00509141
V288	0,005056746
V256	0,004852692
V141	0,004630254
V252	0,004577537
V60	0,004483806
V277	0,004469119
V196	0,004299687
V149	0,004286119
V249	0,004283806
V269	0,004229373
V340	0,004102403
V68	0,004079627
V129	0,004068716
V182	0,004065896
V175	0,004022313
V156	0,004015923
V245	0,003971388
V159	0,003909908
V54	0,003896582
V145	0,003891578
V98	0,003816439
V302	0,003778284
V99	0,003772463
V323	0,003681597
V27	0,003675284
V194	0,003616104
V112	0,003598627
V231	0,003561106
V266	0,003537179
V233	0,003535104
V166	0,00351102
V164	0,003436846
V151	0,003435033
V360	0,003414612
V157	0,003410476
V272	0,003410439
V319	0,003383836
V128	0,003381909
V206	0,003242328
V97	0,003222045
V290	0,003214627
V320	0,003207478
V132	0,003063925
V332	0,003061731
V313	0,003015418
V282	0,003011716
V212	0,003009358
V176	0,003005837

V303	0,002970179
V279	0,002967667
V202	0,002905582
V224	0,002812164
V155	0,002788161
V134	0,002763463
V216	0,00275509
V190	0,002705193
V349	0,002683821
V167	0,002661746
V308	0,002629985
V304	0,002625403
V154	0,002617239
V19	0,002588254
V69	0,002577642
V214	0,002567104
V106	0,002563493
V172	0,002557063
V96	0,00255103
V102	0,002532292
V275	0,002530955
V222	0,002517104
V301	0,002440537
V193	0,002417424
V75	0,002404015
V240	0,002397627
V263	0,002371955
V170	0,002356968
V192	0,002356594
V353	0,002300269
V237	0,002293403
V307	0,002285642
V65	0,002275567
V26	0,002239328
V101	0,002238909
V356	0,002222552
V250	0,002219507
V191	0,002213836
V296	0,002178851
V125	0,002174894
V235	0,002166955
V210	0,002134075
V36	0,002121149
V343	0,002113522
V221	0,002113373
V107	0,002105576
V4	0,002102821
V246	0,002102179
V17	0,002084507
V243	0,002064119
V74	0,002062815

V261	0,002026791
V10	0,002017239
V122	0,002010578
V292	0,002003701
V291	0,001960776
V89	0,001930215
V285	0,001929866
V139	0,001922459
V117	0,001920226
V284	0,001904738
V316	0,001904567
V371	0,001903731
V14	0,001897179
V179	0,0018827
V329	0,001878194
V289	0,001877716
V105	0,001876149
V270	0,001868317
V327	0,001863343
V211	0,001862776
V161	0,001856836
V9	0,001849209
V109	0,001830731
V46	0,001817507
V217	0,001757582
V153	0,00175573
V95	0,001754522
V171	0,001723649
V152	0,001722136
V34	0,001710985
V335	0,001685821
V341	0,001661388
V352	0,001652791
V59	0,001636343
V369	0,001626776
V103	0,001617894
V253	0,001589485
V280	0,001585635
V25	0,001526284
V295	0,001524701
V283	0,001492507
V357	0,001486687
V119	0,001482698
V124	0,001478339
V116	0,001476984
V72	0,001470179
V257	0,001450625
V242	0,001444328
V260	0,001403791
V265	0,001395563
V298	0,001385149

V78	0,001381597
V363	0,001362015
V339	0,001357433
V51	0,001349791
V204	0,001327881
V43	0,001320672
V223	0,001318
V120	0,001314597
V147	0,001313689
V118	0,001313687
V87	0,0013072
V178	0,001301365
V287	0,001297373
V8	0,001277239
V67	0,001276433
V315	0,001272194
V347	0,001268015
V158	0,001250365
V52	0,001243328
V358	0,001234567
V44	0,001233164
V111	0,001229277
V24	0,001228925
V293	0,001209299
V163	0,001199563
V90	0,001189606
V130	0,001183938
V218	0,001166791
V333	0,001155791
V376	0,0011523
V337	0,001149567
V318	0,001125612
V104	0,001123923
V173	0,001103661
V274	0,001102328
V271	0,001097226
V53	0,001096627
V278	0,001092864
V364	0,001088209
V186	0,001088067
V336	0,001084194
V133	0,001077881
V372	0,001042015
V165	0,001038082
V187	0,001036386
V37	0,001033567
V23	0,00102894
V300	0,000991448
V312	0,000984806
V92	0,000971761
V137	0,000965833

V351	0,000958627
V264	0,000957561
V328	0,000940045
V188	0,00093307
V331	0,000909269
V359	0,000904687
V317	0,000894209
V258	0,000886609
V7	0,000877373
V200	0,00086997
V219	0,000854149
V367	0,000851866
V16	0,000850358
V366	0,00084209
V230	0,000831727
V40	0,000828403
V135	0,000828394
V254	0,000823439
V108	0,00081494
V31	0,000812597
V131	0,000806864
V334	0,000792194
V238	0,000780254
V189	0,00077331
V281	0,00075977
V294	0,000739776
V180	0,000738962
V199	0,00071046
V1	0,000708079
V6	0,000699433
V297	0,000677448
V56	0,000675657
V314	0,000666746
V110	0,000647582
V236	0,000642373
V201	0,000641045
V220	0,000628149
V345	0,000619493
V63	0,000601522
V2	0,000586407
V32	0,000572343
V375	0,000571269
V136	0,000564641
V168	0,000547119
V370	0,000532716
V123	0,000528283
V247	0,000526
V57	0,000525328
V38	0,000524015
V213	0,000513925
V361	0,000510896

V79	0,000506692
V5	0,000495239
V94	0,000490094
V346	0,000485716
V174	0,000475324
V49	0,000462642
V138	0,000458215
V248	0,000451924
V368	0,00045106
V12	0,000447537
V342	0,000439358
V55	0,000427701
V11	0,000424478
V13	0,00040697
V251	0,000401875
V268	0,000401046
V181	0,000396873
V20	0,000386209
V350	0,000384328
V239	0,000382373
V325	0,000371015
V41	0,000368463
V311	0,000360343
V45	0,000351433
V15	0,000342403
V322	0,000340925
V338	0,000338284
V62	0,000311803
V198	0,00031005
V326	0,000302985
V42	0,000291746
V22	0,00026097
V21	0,00025609
V203	0,000251859
V354	0,000249015
V113	0,000247969
V33	0,000247522
V18	0,00023894
V115	0,000235651
V362	0,000232254
V374	0,000225657
V58	0,000224701
V39	0,000223612
V255	0,000198175
V47	0,000193657
V29	0,000184582
V80	0,000164833
V373	0,000162627
V73	0,000153303
V229	0,000148636
V88	0,000147197

V3	0,000125254
V81	0,000122062
V30	0,000119552
V299	0,000116507
V50	0,000100985
V259	8,52727E-05
V348	7,94328E-05
V140	7,82459E-05
V66	6,21343E-05
V305	6,05672E-05
V48	5,87015E-05
V344	4,63731E-05
V321	3,61343E-05
V234	3,58788E-05
V146	2,98448E-05
V93	1,78308E-05
V114	1,77627E-05
V121	0
V306	0
V241	0

R-code and output for comparison of demographic and clinical data between individual train and validation data sets

```
#load data
Train_Test<-read.xlsx(xlsxFile = "train_test_assignment.xlsx", sheet = "Tabelle1",
                      colNames = TRUE)

#perform t-test for UPDRS for all train:validation splits
group_vars<-colnames(Train_Test[,2:68])
grepl("^run_",colnames(Train_Test))
##  [1] FALSE  TRUE  TRUE  TRUE  TRUE  TRUE  TRUE  TRUE  TRUE  TRUE  TRUE  TRUE
## [13]  TRUE  TRUE  TRUE  TRUE  TRUE  TRUE  TRUE  TRUE  TRUE  TRUE  TRUE  TRUE
## [25]  TRUE  TRUE  TRUE  TRUE  TRUE  TRUE  TRUE  TRUE  TRUE  TRUE  TRUE  TRUE
## [37]  TRUE  TRUE  TRUE  TRUE  TRUE  TRUE  TRUE  TRUE  TRUE  TRUE  TRUE  TRUE
## [49]  TRUE  TRUE  TRUE  TRUE  TRUE  TRUE  TRUE  TRUE  TRUE  TRUE  TRUE  TRUE
## [61]  TRUE  TRUE  TRUE  TRUE  TRUE  TRUE  TRUE  TRUE FALSE FALSE
results<-lapply(group_vars,
                function(group_var){
                  data_filtered<-na.omit(Train_Test[c("UPDRS",group_var)])#NA remove
                  t_test<-t.test(formula=as.formula(paste("UPDRS~",group_var)), data=data_filtered)
result<-list(Group=group_var,
             Statistic=t_test$statistic,
             p_value=t_test$p.value,
            mean=t_test$estimate)
return(result)
})

print(results)
## [[1]]
## [[1]]$Group
## [1] "run_1"
##
## [[1]]$Statistic
##         t
```

```
## -0.9496978
##
## [[1]]$p_value
## [1] 0.3515293
##
## [[1]]$mean
## mean in group 0 mean in group 1
##        23.06667        25.94444
##
##
## [[2]]
## [[2]]$Group
## [1] "run_2"
##
## [[2]]$Statistic
##          t
## -0.9496978
##
## [[2]]$p_value
## [1] 0.3515293
##
## [[2]]$mean
## mean in group 0 mean in group 1
##        23.06667        25.94444
##
##
## [[3]]
## [[3]]$Group
## [1] "run_3"
##
## [[3]]$Statistic
##          t
## -0.9496978
```

```
##
## [[3]]$p_value
## [1] 0.3515293
##
## [[3]]$mean
## mean in group 0 mean in group 1
##        23.06667        25.94444
##
##
## [[4]]
## [[4]]$Group
## [1] "run_4"
##
## [[4]]$Statistic
##          t
## -0.9496978
##
## [[4]]$p_value
## [1] 0.3515293
##
## [[4]]$mean
## mean in group 0 mean in group 1
##        23.06667        25.94444
##
##
## [[5]]
## [[5]]$Group
## [1] "run_5"
##
## [[5]]$Statistic
##          t
## -0.9496978
##
```

```
## [[5]]$p_value
## [1] 0.3515293
##
## [[5]]$mean
## mean in group 0 mean in group 1
##        23.06667        25.94444
##
##
## [[6]]
## [[6]]$Group
## [1] "run_6"
##
## [[6]]$Statistic
##          t
## -0.9496978
##
## [[6]]$p_value
## [1] 0.3515293
##
## [[6]]$mean
## mean in group 0 mean in group 1
##        23.06667        25.94444
##
##
## [[7]]
## [[7]]$Group
## [1] "run_7"
##
## [[7]]$Statistic
##          t
## -0.9496978
##
## [[7]]$p_value
```

```
## [1] 0.3515293
##
## [[7]]$mean
## mean in group 0 mean in group 1
##        23.06667        25.94444
##
##
## [[8]]
## [[8]]$Group
## [1] "run_8"
##
## [[8]]$Statistic
##          t
## -0.9496978
##
## [[8]]$p_value
## [1] 0.3515293
##
## [[8]]$mean
## mean in group 0 mean in group 1
##        23.06667        25.94444
##
##
## [[9]]
## [[9]]$Group
## [1] "run_9"
##
## [[9]]$Statistic
##          t
## -0.9496978
##
## [[9]]$p_value
## [1] 0.3515293
```

```
##
## [[9]]$mean
## mean in group 0 mean in group 1
##        23.06667        25.94444
##
##
## [[10]]
## [[10]]$Group
## [1] "run_10"
##
## [[10]]$Statistic
##          t
## -0.9496978
##
## [[10]]$p_value
## [1] 0.3515293
##
## [[10]]$mean
## mean in group 0 mean in group 1
##        23.06667        25.94444
##
##
## [[11]]
## [[11]]$Group
## [1] "run_11"
##
## [[11]]$Statistic
##          t
## -0.9496978
##
## [[11]]$p_value
## [1] 0.3515293
##
```

```
## [[11]]$mean
## mean in group 0 mean in group 1
##        23.06667        25.94444
##
##
## [[12]]
## [[12]]$Group
## [1] "run_12"
##
## [[12]]$Statistic
##          t
## -0.9496978
##
## [[12]]$p_value
## [1] 0.3515293
##
## [[12]]$mean
## mean in group 0 mean in group 1
##        23.06667        25.94444
##
##
## [[13]]
## [[13]]$Group
## [1] "run_13"
##
## [[13]]$Statistic
##          t
## -0.9496978
##
## [[13]]$p_value
## [1] 0.3515293
##
## [[13]]$mean
```

```
## mean in group 0 mean in group 1
##        23.06667        25.94444
##
##
## [[14]]
## [[14]]$Group
## [1] "run_14"
##
## [[14]]$Statistic
##          t
## -0.9496978
##
## [[14]]$p_value
## [1] 0.3515293
##
## [[14]]$mean
## mean in group 0 mean in group 1
##        23.06667        25.94444
##
##
## [[15]]
## [[15]]$Group
## [1] "run_15"
##
## [[15]]$Statistic
##          t
## -0.9496978
##
## [[15]]$p_value
## [1] 0.3515293
##
## [[15]]$mean
## mean in group 0 mean in group 1
```

```
##        23.06667        25.94444
##
##
## [[16]]
## [[16]]$Group
## [1] "run_16"
##
## [[16]]$Statistic
##          t
## -0.9496978
##
## [[16]]$p_value
## [1] 0.3515293
##
## [[16]]$mean
## mean in group 0 mean in group 1
##        23.06667        25.94444
##
##
## [[17]]
## [[17]]$Group
## [1] "run_17"
##
## [[17]]$Statistic
##          t
## -0.8101183
##
## [[17]]$p_value
## [1] 0.4257469
##
## [[17]]$mean
## mean in group 0 mean in group 1
##        23.06667        25.51429
```

```
##
##
## [[18]]
## [[18]]$Group
## [1] "run_18"
##
## [[18]]$Statistic
##          t
## -0.8018436
##
## [[18]]$p_value
## [1] 0.4304645
##
## [[18]]$mean
## mean in group 0 mean in group 1
##        23.06667        25.48571
##
##
## [[19]]
## [[19]]$Group
## [1] "run_19"
##
## [[19]]$Statistic
##          t
## -0.8268336
##
## [[19]]$p_value
## [1] 0.4163206
##
## [[19]]$mean
## mean in group 0 mean in group 1
##        23.06667        25.57143
##
```

```
##
## [[20]]
## [[20]]$Group
## [1] "run_20"
##
## [[20]]$Statistic
##          t
## -0.9703736
##
## [[20]]$p_value
## [1] 0.3411278
##
## [[20]]$mean
## mean in group 0 mean in group 1
##        23.06667        26.02857
##
##
## [[21]]
## [[21]]$Group
## [1] "run_21"
##
## [[21]]$Statistic
##          t
## -0.8523498
##
## [[21]]$p_value
## [1] 0.4021988
##
## [[21]]$mean
## mean in group 0 mean in group 1
##        23.06667        25.65714
##
##
```

```
## [[22]]
## [[22]]$Group
## [1] "run_22"
##
## [[22]]$Statistic
##          t
## -0.9898193
##
## [[22]]$p_value
## [1] 0.3317399
##
## [[22]]$mean
## mean in group 0 mean in group 1
##        23.06667        26.08571
##
##
## [[23]]
## [[23]]$Group
## [1] "run_23"
##
## [[23]]$Statistic
##         t
## -0.891398
##
## [[23]]$p_value
## [1] 0.381085
##
## [[23]]$mean
## mean in group 0 mean in group 1
##        23.40000        26.08571
##
##
## [[24]]
```

```
## [[24]]$Group
## [1] "run_24"
##
## [[24]]$Statistic
##          t
## -0.9335367
##
## [[24]]$p_value
## [1] 0.3594618
##
## [[24]]$mean
## mean in group 0 mean in group 1
##            23.4            26.2
##
##
## [[25]]
## [[25]]$Group
## [1] "run_25"
##
## [[25]]$Statistic
##          t
## -0.9503132
##
## [[25]]$p_value
## [1] 0.3511013
##
## [[25]]$mean
## mean in group 0 mean in group 1
##        23.33333        26.20000
##
##
## [[26]]
## [[26]]$Group
```

```
## [1] "run_26"
##
## [[26]]$Statistic
##          t
## -0.8493015
##
## [[26]]$p_value
## [1] 0.4036317
##
## [[26]]$mean
## mean in group 0 mean in group 1
##        23.33333        25.91429
##
##
## [[27]]
## [[27]]$Group
## [1] "run_27"
##
## [[27]]$Statistic
##          t
## -0.7568192
##
## [[27]]$p_value
## [1] 0.456152
##
## [[27]]$mean
## mean in group 0 mean in group 1
##        23.33333        25.62857
##
##
## [[28]]
## [[28]]$Group
## [1] "run_28"
```

```
##
## [[28]]$Statistic
##          t
## -0.7838309
##
## [[28]]$p_value
## [1] 0.4403833
##
## [[28]]$mean
## mean in group 0 mean in group 1
##        23.33333        25.71429
##
##
## [[29]]
## [[29]]$Group
## [1] "run_29"
##
## [[29]]$Statistic
##         t
## -0.811441
##
## [[29]]$p_value
## [1] 0.424629
##
## [[29]]$mean
## mean in group 0 mean in group 1
##        23.33333        25.80000
##
##
## [[30]]
## [[30]]$Group
## [1] "run_30"
##
```

```
## [[30]]$Statistic
##          t
## -0.8493015
##
## [[30]]$p_value
## [1] 0.4036317
##
## [[30]]$mean
## mean in group 0 mean in group 1
##        23.33333        25.91429
##
##
## [[31]]
## [[31]]$Group
## [1] "run_31"
##
## [[31]]$Statistic
##          t
## -0.8207887
##
## [[31]]$p_value
## [1] 0.4193794
##
## [[31]]$mean
## mean in group 0 mean in group 1
##        23.33333        25.82857
##
##
## [[32]]
## [[32]]$Group
## [1] "run_32"
##
## [[32]]$Statistic
```

```
##          t
## -0.8785768
##
## [[32]]$p_value
## [1] 0.3878815
##
## [[32]]$mean
## mean in group 0 mean in group 1
##        23.33333        26.00000
##
##
## [[33]]
## [[33]]$Group
## [1] "run_33"
##
## [[33]]$Statistic
##          t
## -0.8493015
##
## [[33]]$p_value
## [1] 0.4036317
##
## [[33]]$mean
## mean in group 0 mean in group 1
##        23.33333        25.91429
##
##
## [[34]]
## [[34]]$Group
## [1] "run_34"
##
## [[34]]$Statistic
##          t
```

```
## -0.8302129
##
## [[34]]$p_value
## [1] 0.4141301
##
## [[34]]$mean
## mean in group 0 mean in group 1
##        23.33333        25.85714
##
##
## [[35]]
## [[35]]$Group
## [1] "run_35"
##
## [[35]]$Statistic
##          t
## -0.8589714
##
## [[35]]$p_value
## [1] 0.3983821
##
## [[35]]$mean
## mean in group 0 mean in group 1
##        23.33333        25.94286
##
##
## [[36]]
## [[36]]$Group
## [1] "run_36"
##
## [[36]]$Statistic
##        t
## -0.71296
```

```
##
## [[36]]$p_value
## [1] 0.4824931
##
## [[36]]$mean
## mean in group 0 mean in group 1
##        23.33333        25.48571
##
##
## [[37]]
## [[37]]$Group
## [1] "run_37"
##
## [[37]]$Statistic
##          t
## -0.8207887
##
## [[37]]$p_value
## [1] 0.4193794
##
## [[37]]$mean
## mean in group 0 mean in group 1
##        23.33333        25.82857
##
##
## [[38]]
## [[38]]$Group
## [1] "run_38"
##
## [[38]]$Statistic
##          t
## -0.6619129
##
```

```
## [[38]]$p_value
## [1] 0.5142733
##
## [[38]]$mean
## mean in group 0 mean in group 1
##        23.33333        25.31429
##
##
## [[39]]
## [[39]]$Group
## [1] "run_39"
##
## [[39]]$Statistic
##          t
## -0.2167221
##
## [[39]]$p_value
## [1] 0.8304279
##
## [[39]]$mean
## mean in group 0 mean in group 1
##        24.60000        25.31429
##
##
## [[40]]
## [[40]]$Group
## [1] "run_40"
##
## [[40]]$Statistic
##          t
## -0.9464662
##
## [[40]]$p_value
```

```
## [1] 0.3519478
##
## [[40]]$mean
## mean in group 0 mean in group 1
##        22.80000        25.31429
##
##
## [[41]]
## [[41]]$Group
## [1] "run_41"
##
## [[41]]$Statistic
##         t
## -1.254388
##
## [[41]]$p_value
## [1] 0.2190071
##
## [[41]]$mean
## mean in group 0 mean in group 1
##        22.80000        26.25714
##
##
## [[42]]
## [[42]]$Group
## [1] "run_42"
##
## [[42]]$Statistic
##         t
## -1.199449
##
## [[42]]$p_value
## [1] 0.2393132
```

```
##
## [[42]]$mean
## mean in group 0 mean in group 1
##        22.80000        26.11429
##
##
## [[43]]
## [[43]]$Group
## [1] "run_43"
##
## [[43]]$Statistic
##         t
## -1.254388
##
## [[43]]$p_value
## [1] 0.2190071
##
## [[43]]$mean
## mean in group 0 mean in group 1
##        22.80000        26.25714
##
##
## [[44]]
## [[44]]$Group
## [1] "run_44"
##
## [[44]]$Statistic
##        t
## -1.12836
##
## [[44]]$p_value
## [1] 0.2677282
##
```

```
## [[44]]$mean
## mean in group 0 mean in group 1
##        22.80000        25.91429
##
##
## [[45]]
## [[45]]$Group
## [1] "run_45"
##
## [[45]]$Statistic
##         t
## -1.178487
##
## [[45]]$p_value
## [1] 0.247438
##
## [[45]]$mean
## mean in group 0 mean in group 1
##        22.80000        26.05714
##
##
## [[46]]
## [[46]]$Group
## [1] "run_46"
##
## [[46]]$Statistic
##        t
## -1.03376
##
## [[46]]$p_value
## [1] 0.3091283
##
## [[46]]$mean
```

```
## mean in group 0 mean in group 1
##        23.20000        26.05714
##
##
## [[47]]
## [[47]]$Group
## [1] "run_47"
##
## [[47]]$Statistic
##         t
## -1.090637
##
## [[47]]$p_value
## [1] 0.2838173
##
## [[47]]$mean
## mean in group 0 mean in group 1
##            23.2            26.2
##
##
## [[48]]
## [[48]]$Group
## [1] "run_48"
##
## [[48]]$Statistic
##         t
## -1.588281
##
## [[48]]$p_value
## [1] 0.1219741
##
## [[48]]$mean
## mean in group 0 mean in group 1
```

```
##        21.93333        26.20000
##
##
## [[49]]
## [[49]]$Group
## [1] "run_49"
##
## [[49]]$Statistic
##         t
## -1.284265
##
## [[49]]$p_value
## [1] 0.2088205
##
## [[49]]$mean
## mean in group 0 mean in group 1
##            22.6            26.2
##
##
## [[50]]
## [[50]]$Group
## [1] "run_50"
##
## [[50]]$Statistic
##         t
## -1.083518
##
## [[50]]$p_value
## [1] 0.2876996
##
## [[50]]$mean
## mean in group 0 mean in group 1
##        22.60000        25.57143
```

```
##
##
## [[51]]
## [[51]]$Group
## [1] "run_51"
##
## [[51]]$Statistic
##         t
## -1.447478
##
## [[51]]$p_value
## [1] 0.1584858
##
## [[51]]$mean
## mean in group 0 mean in group 1
##            22.6            26.6
##
##
## [[52]]
## [[52]]$Group
## [1] "run_52"
##
## [[52]]$Statistic
##         t
## -1.176943
##
## [[52]]$p_value
## [1] 0.2485199
##
## [[52]]$mean
## mean in group 0 mean in group 1
##        22.60000        25.88571
##
```

```
##
## [[53]]
## [[53]]$Group
## [1] "run_53"
##
## [[53]]$Statistic
##         t
## -1.316425
##
## [[53]]$p_value
## [1] 0.1979742
##
## [[53]]$mean
## mean in group 0 mean in group 1
##        22.60000        26.28571
##
##
## [[54]]
## [[54]]$Group
## [1] "run_54"
##
## [[54]]$Statistic
##         t
## -1.361538
##
## [[54]]$p_value
## [1] 0.1835425
##
## [[54]]$mean
## mean in group 0 mean in group 1
##            22.6            26.4
##
##
```

```
## [[55]]
## [[55]]$Group
## [1] "run_55"
##
## [[55]]$Statistic
##         t
## -1.243419
##
## [[55]]$p_value
## [1] 0.2232845
##
## [[55]]$mean
## mean in group 0 mean in group 1
##        22.60000        26.08571
##
##
## [[56]]
## [[56]]$Group
## [1] "run_56"
##
## [[56]]$Statistic
##         t
## -1.069417
##
## [[56]]$p_value
## [1] 0.2934495
##
## [[56]]$mean
## mean in group 0 mean in group 1
##        23.06667        26.08571
##
##
## [[57]]
```

```
## [[57]]$Group
## [1] "run_57"
##
## [[57]]$Statistic
##          t
## -0.8554871
##
## [[57]]$p_value
## [1] 0.3985327
##
## [[57]]$mean
## mean in group 0 mean in group 1
##        23.80000        26.08571
##
##
## [[58]]
## [[58]]$Group
## [1] "run_58"
##
## [[58]]$Statistic
##         t
## -1.243419
##
## [[58]]$p_value
## [1] 0.2232845
##
## [[58]]$mean
## mean in group 0 mean in group 1
##        22.60000        26.08571
##
##
## [[59]]
## [[59]]$Group
```

```
## [1] "run_59"
##
## [[59]]$Statistic
##         t
## -1.385132
##
## [[59]]$p_value
## [1] 0.1763506
##
## [[59]]$mean
## mean in group 0 mean in group 1
##        22.60000        26.45714
##
##
## [[60]]
## [[60]]$Group
## [1] "run_60"
##
## [[60]]$Statistic
##         t
## -1.551455
##
## [[60]]$p_value
## [1] 0.131662
##
## [[60]]$mean
## mean in group 0 mean in group 1
##        22.06667        26.45714
##
##
## [[61]]
## [[61]]$Group
## [1] "run_61"
```

```
##
## [[61]]$Statistic
##         t
## -1.595537
##
## [[61]]$p_value
## [1] 0.1215288
##
## [[61]]$mean
## mean in group 0 mean in group 1
##        22.06667        26.57143
##
##
## [[62]]
## [[62]]$Group
## [1] "run_62"
##
## [[62]]$Statistic
##         t
## -1.667534
##
## [[62]]$p_value
## [1] 0.1064818
##
## [[62]]$mean
## mean in group 0 mean in group 1
##        22.06667        26.74286
##
##
## [[63]]
## [[63]]$Group
## [1] "run_63"
##
```

```
## [[63]]$Statistic
##         t
## -1.815975
##
## [[63]]$p_value
## [1] 0.08036794
##
## [[63]]$mean
## mean in group 0 mean in group 1
##        21.53333        26.74286
##
##
## [[64]]
## [[64]]$Group
## [1] "run_64"
##
## [[64]]$Statistic
##         t
## -1.733752
##
## [[64]]$p_value
## [1] 0.09414349
##
## [[64]]$mean
## mean in group 0 mean in group 1
##        21.80000        26.74286
##
##
## [[65]]
## [[65]]$Group
## [1] "run_65"
##
## [[65]]$Statistic
```

```
##         t
## -1.677436
##
## [[65]]$p_value
## [1] 0.104648
##
## [[65]]$mean
## mean in group 0 mean in group 1
##            21.8            26.6
##
##
## [[66]]
## [[66]]$Group
## [1] "run_66"
##
## [[66]]$Statistic
##         t
## -1.780317
##
## [[66]]$p_value
## [1] 0.08585524
##
## [[66]]$mean
## mean in group 0 mean in group 1
##        21.53333        26.60000
##
##
## [[67]]
## [[67]]$Group
## [1] "run_67"
##
## [[67]]$Statistic
##         t
```

```
## -1.554003
##
## [[67]]$p_value
## [1] 0.13131
##
## [[67]]$mean
## mean in group 0 mean in group 1
##            22.2            26.6
#perform t-tests for Age for all train:validation splits
group_vars<-colnames(Train_Test[,2:68])
grepl("^run_",colnames(Train_Test))
##  [1] FALSE  TRUE  TRUE  TRUE  TRUE  TRUE  TRUE  TRUE  TRUE  TRUE  TRUE  TRUE
## [13]  TRUE  TRUE  TRUE  TRUE  TRUE  TRUE  TRUE  TRUE  TRUE  TRUE  TRUE  TRUE
## [25]  TRUE  TRUE  TRUE  TRUE  TRUE  TRUE  TRUE  TRUE  TRUE  TRUE  TRUE  TRUE
## [37]  TRUE  TRUE  TRUE  TRUE  TRUE  TRUE  TRUE  TRUE  TRUE  TRUE  TRUE  TRUE
## [49]  TRUE  TRUE  TRUE  TRUE  TRUE  TRUE  TRUE  TRUE  TRUE  TRUE  TRUE  TRUE
## [61]  TRUE  TRUE  TRUE  TRUE  TRUE  TRUE  TRUE  TRUE FALSE FALSE
results<-lapply(group_vars,
                function(group_var){
                  data_filtered<-na.omit(Train_Test[c("Age",group_var)])#NA remove
                  t_test<-t.test(formula=as.formula(paste("Age~",group_var)), data=data_filtered)
                  result<-list(Group=group_var,
                               Statistic=t_test$statistic,
                               p_value=t_test$p.value)
                  return(result)
                })

print(results)
## [[1]]
## [[1]]$Group
## [1] "run_1"
##
## [[1]]$Statistic
```

```
##          t
## -0.4857734
##
## [[1]]$p_value
## [1] 0.6306623
##
##
## [[2]]
## [[2]]$Group
## [1] "run_2"
##
## [[2]]$Statistic
##          t
## -0.5630284
##
## [[2]]$p_value
## [1] 0.5775747
##
##
## [[3]]
## [[3]]$Group
## [1] "run_3"
##
## [[3]]$Statistic
##          t
## -0.5895442
##
## [[3]]$p_value
## [1] 0.5598945
##
##
## [[4]]
## [[4]]$Group
```

```
## [1] "run_4"
##
## [[4]]$Statistic
##          t
## -0.9447967
##
## [[4]]$p_value
## [1] 0.3520106
##
##
## [[5]]
## [[5]]$Group
## [1] "run_5"
##
## [[5]]$Statistic
##         t
## -1.065991
##
## [[5]]$p_value
## [1] 0.2944596
##
##
## [[6]]
## [[6]]$Group
## [1] "run_6"
##
## [[6]]$Statistic
##         t
## -1.275566
##
## [[6]]$p_value
## [1] 0.2111727
##
```

```
##
## [[7]]
## [[7]]$Group
## [1] "run_7"
##
## [[7]]$Statistic
##         t
## -1.108171
##
## [[7]]$p_value
## [1] 0.2760986
##
##
## [[8]]
## [[8]]$Group
## [1] "run_8"
##
## [[8]]$Statistic
##        t
## -1.09914
##
## [[8]]$p_value
## [1] 0.2799574
##
##
## [[9]]
## [[9]]$Group
## [1] "run_9"
##
## [[9]]$Statistic
##         t
## -1.108171
##
```

```
## [[9]]$p_value
## [1] 0.2760986
##
##
## [[10]]
## [[10]]$Group
## [1] "run_10"
##
## [[10]]$Statistic
##         t
## -1.267416
##
## [[10]]$p_value
## [1] 0.2145848
##
##
## [[11]]
## [[11]]$Group
## [1] "run_11"
##
## [[11]]$Statistic
##         t
## -1.072595
##
## [[11]]$p_value
## [1] 0.2915353
##
##
## [[12]]
## [[12]]$Group
## [1] "run_12"
##
## [[12]]$Statistic
```

```
##         t
## -1.135843
##
## [[12]]$p_value
## [1] 0.2645256
##
##
## [[13]]
## [[13]]$Group
## [1] "run_13"
##
## [[13]]$Statistic
##       t
## -1.0384
##
## [[13]]$p_value
## [1] 0.3069707
##
##
## [[14]]
## [[14]]$Group
## [1] "run_14"
##
## [[14]]$Statistic
##         t
## -1.235173
##
## [[14]]$p_value
## [1] 0.2260595
##
##
## [[15]]
## [[15]]$Group
```

```
## [1] "run_15"
##
## [[15]]$Statistic
##         t
## -1.220854
##
## [[15]]$p_value
## [1] 0.2313097
##
##
## [[16]]
## [[16]]$Group
## [1] "run_16"
##
## [[16]]$Statistic
##         t
## -1.297304
##
## [[16]]$p_value
## [1] 0.204268
##
##
## [[17]]
## [[17]]$Group
## [1] "run_17"
##
## [[17]]$Statistic
##         t
## -1.143268
##
## [[17]]$p_value
## [1] 0.2617762
##
```

```
##
## [[18]]
## [[18]]$Group
## [1] "run_18"
##
## [[18]]$Statistic
##         t
## -1.392764
##
## [[18]]$p_value
## [1] 0.1741566
##
##
## [[19]]
## [[19]]$Group
## [1] "run_19"
##
## [[19]]$Statistic
##         t
## -1.176919
##
## [[19]]$p_value
## [1] 0.2482331
##
##
## [[20]]
## [[20]]$Group
## [1] "run_20"
##
## [[20]]$Statistic
##         t
## -1.297304
##
```

```
## [[20]]$p_value
## [1] 0.204268
##
##
## [[21]]
## [[21]]$Group
## [1] "run_21"
##
## [[21]]$Statistic
##         t
## -1.212059
##
## [[21]]$p_value
## [1] 0.2346843
##
##
## [[22]]
## [[22]]$Group
## [1] "run_22"
##
## [[22]]$Statistic
##         t
## -1.151548
##
## [[22]]$p_value
## [1] 0.2583917
##
##
## [[23]]
## [[23]]$Group
## [1] "run_23"
##
## [[23]]$Statistic
```

```
##          t
## -0.9918137
##
## [[23]]$p_value
## [1] 0.3291615
##
##
## [[24]]
## [[24]]$Group
## [1] "run_24"
##
## [[24]]$Statistic
##         t
## -1.074743
##
## [[24]]$p_value
## [1] 0.2911039
##
##
## [[25]]
## [[25]]$Group
## [1] "run_25"
##
## [[25]]$Statistic
##         t
## -1.618458
##
## [[25]]$p_value
## [1] 0.1155868
##
##
## [[26]]
## [[26]]$Group
```

```
## [1] "run_26"
##
## [[26]]$Statistic
##         t
## -1.657101
##
## [[26]]$p_value
## [1] 0.1073297
##
##
## [[27]]
## [[27]]$Group
## [1] "run_27"
##
## [[27]]$Statistic
##         t
## -1.596839
##
## [[27]]$p_value
## [1] 0.1204677
##
##
## [[28]]
## [[28]]$Group
## [1] "run_28"
##
## [[28]]$Statistic
##         t
## -1.804915
##
## [[28]]$p_value
## [1] 0.08057144
##
```

```
##
## [[29]]
## [[29]]$Group
## [1] "run_29"
##
## [[29]]$Statistic
##         t
## -1.665232
##
## [[29]]$p_value
## [1] 0.1056645
##
##
## [[30]]
## [[30]]$Group
## [1] "run_30"
##
## [[30]]$Statistic
##         t
## -1.754449
##
## [[30]]$p_value
## [1] 0.08891229
##
##
## [[31]]
## [[31]]$Group
## [1] "run_31"
##
## [[31]]$Statistic
##         t
## -1.625923
##
```

```
## [[31]]$p_value
## [1] 0.1139463
##
##
## [[32]]
## [[32]]$Group
## [1] "run_32"
##
## [[32]]$Statistic
##         t
## -1.726117
##
## [[32]]$p_value
## [1] 0.09394275
##
##
## [[33]]
## [[33]]$Group
## [1] "run_33"
##
## [[33]]$Statistic
##         t
## -1.815522
##
## [[33]]$p_value
## [1] 0.07891457
##
##
## [[34]]
## [[34]]$Group
## [1] "run_34"
##
## [[34]]$Statistic
```

```
##         t
## -1.735405
##
## [[34]]$p_value
## [1] 0.092265
##
##
## [[35]]
## [[35]]$Group
## [1] "run_35"
##
## [[35]]$Statistic
##        t
## -1.71698
##
## [[35]]$p_value
## [1] 0.09562073
##
##
## [[36]]
## [[36]]$Group
## [1] "run_36"
##
## [[36]]$Statistic
##        t
## -1.71698
##
## [[36]]$p_value
## [1] 0.09562073
##
##
## [[37]]
## [[37]]$Group
```

```
## [1] "run_37"
##
## [[37]]$Statistic
##         t
## -1.649106
##
## [[37]]$p_value
## [1] 0.1089909
##
##
## [[38]]
## [[38]]$Group
## [1] "run_38"
##
## [[38]]$Statistic
##         t
## -1.699155
##
## [[38]]$p_value
## [1] 0.09897531
##
##
## [[39]]
## [[39]]$Group
## [1] "run_39"
##
## [[39]]$Statistic
##         t
## -1.458837
##
## [[39]]$p_value
## [1] 0.1543962
##
```

```
##
## [[40]]
## [[40]]$Group
## [1] "run_40"
##
## [[40]]$Statistic
##        t
## -1.51525
##
## [[40]]$p_value
## [1] 0.1396107
##
##
## [[41]]
## [[41]]$Group
## [1] "run_41"
##
## [[41]]$Statistic
##        t
## -1.51525
##
## [[41]]$p_value
## [1] 0.1396107
##
##
## [[42]]
## [[42]]$Group
## [1] "run_42"
##
## [[42]]$Statistic
##         t
## -1.432816
##
```

```
## [[42]]$p_value
## [1] 0.1616782
##
##
## [[43]]
## [[43]]$Group
## [1] "run_43"
##
## [[43]]$Statistic
##         t
## -1.535037
##
## [[43]]$p_value
## [1] 0.1347235
##
##
## [[44]]
## [[44]]$Group
## [1] "run_44"
##
## [[44]]$Statistic
##         t
## -1.344964
##
## [[44]]$p_value
## [1] 0.1884498
##
##
## [[45]]
## [[45]]$Group
## [1] "run_45"
##
## [[45]]$Statistic
```

```
##        t
## -1.48661
##
## [[45]]$p_value
## [1] 0.1469601
##
##
## [[46]]
## [[46]]$Group
## [1] "run_46"
##
## [[46]]$Statistic
##         t
## -1.782662
##
## [[46]]$p_value
## [1] 0.08379253
##
##
## [[47]]
## [[47]]$Group
## [1] "run_47"
##
## [[47]]$Statistic
##         t
## -1.782662
##
## [[47]]$p_value
## [1] 0.08379253
##
##
## [[48]]
## [[48]]$Group
```

```
## [1] "run_48"
##
## [[48]]$Statistic
##         t
## -1.816568
##
## [[48]]$p_value
## [1] 0.07825508
##
##
## [[49]]
## [[49]]$Group
## [1] "run_49"
##
## [[49]]$Statistic
##         t
## -1.816568
##
## [[49]]$p_value
## [1] 0.07825508
##
##
## [[50]]
## [[50]]$Group
## [1] "run_50"
##
## [[50]]$Statistic
##         t
## -1.912756
##
## [[50]]$p_value
## [1] 0.0642556
##
```

```
##
## [[51]]
## [[51]]$Group
## [1] "run_51"
##
## [[51]]$Statistic
##         t
## -1.912756
##
## [[51]]$p_value
## [1] 0.0642556
##
##
## [[52]]
## [[52]]$Group
## [1] "run_52"
##
## [[52]]$Statistic
##         t
## -1.884487
##
## [[52]]$p_value
## [1] 0.06809641
##
##
## [[53]]
## [[53]]$Group
## [1] "run_53"
##
## [[53]]$Statistic
##         t
## -1.893733
##
```

```
## [[53]]$p_value
## [1] 0.06681641
##
##
## [[54]]
## [[54]]$Group
## [1] "run_54"
##
## [[54]]$Statistic
##         t
## -1.893733
##
## [[54]]$p_value
## [1] 0.06681641
##
##
## [[55]]
## [[55]]$Group
## [1] "run_55"
##
## [[55]]$Statistic
##         t
## -1.857776
##
## [[55]]$p_value
## [1] 0.07192832
##
##
## [[56]]
## [[56]]$Group
## [1] "run_56"
##
## [[56]]$Statistic
```

```
##          t
## -1.828252
##
## [[56]]$p_value
## [1] 0.07639158
##
##
## [[57]]
## [[57]]$Group
## [1] "run_57"
##
## [[57]]$Statistic
##          t
## -1.471261
##
## [[57]]$p_value
## [1] 0.1504291
##
##
## [[58]]
## [[58]]$Group
## [1] "run_58"
##
## [[58]]$Statistic
##          t
## -1.430571
##
## [[58]]$p_value
## [1] 0.1615016
##
##
## [[59]]
## [[59]]$Group
```

```
## [1] "run_59"
##
## [[59]]$Statistic
##        t
## -1.31998
##
## [[59]]$p_value
## [1] 0.195214
##
##
## [[60]]
## [[60]]$Group
## [1] "run_60"
##
## [[60]]$Statistic
##        t
## -1.31998
##
## [[60]]$p_value
## [1] 0.195214
##
##
## [[61]]
## [[61]]$Group
## [1] "run_61"
##
## [[61]]$Statistic
##         t
## -1.430571
##
## [[61]]$p_value
## [1] 0.1615016
##
```

```
##
## [[62]]
## [[62]]$Group
## [1] "run_62"
##
## [[62]]$Statistic
##         t
## -1.340872
##
## [[62]]$p_value
## [1] 0.1884359
##
##
## [[63]]
## [[63]]$Group
## [1] "run_63"
##
## [[63]]$Statistic
##         t
## -1.399631
##
## [[63]]$p_value
## [1] 0.1703155
##
##
## [[64]]
## [[64]]$Group
## [1] "run_64"
##
## [[64]]$Statistic
##         t
## -1.287828
##
```

```
## [[64]]$p_value
## [1] 0.2059874
##
##
## [[65]]
## [[65]]$Group
## [1] "run_65"
##
## [[65]]$Statistic
##         t
## -1.184364
##
## [[65]]$p_value
## [1] 0.243867
##
##
## [[66]]
## [[66]]$Group
## [1] "run_66"
##
## [[66]]$Statistic
##         t
## -1.337988
##
## [[66]]$p_value
## [1] 0.1889425
##
##
## [[67]]
## [[67]]$Group
## [1] "run_67"
##
## [[67]]$Statistic
```

```
##          t
## -0.8951532
##
## [[67]]$p_value
## [1] 0.376144
```
